# Pleomorphic Adenoma of the Nasal Cavity

**DOI:** 10.7759/cureus.65969

**Published:** 2024-08-01

**Authors:** Rehman Basharat, Alexander Bjorling, Ghassan Samara

**Affiliations:** 1 Otolaryngology, Stony Brook University, New York, USA

**Keywords:** tumor, nasal septum, salivary gland tumor, benign nasal mass, pleomorphic adenoma

## Abstract

Pleomorphic adenoma (PA) most commonly manifests in the parotid gland, though it occasionally emerges in atypical locations. We present a case involving an 87-year-old female who exhibited chronic left-sided nasal symptoms, leading to the diagnosis of PA in the nasal cavity. This diagnosis was confirmed through rhinoscopy and subsequent pathological examination following the surgical excision of an 8x8 mm mass. The procedure, which included tumor-based cautery, alleviated her symptoms effectively. A follow-up strategy was established to monitor for any signs of recurrence. The patient has shown no signs of recurrence at subsequent three-month follow-up visits, highlighting the success of the intervention. This case underscores the importance of early recognition and intervention in atypical presentations of PA, which is crucial to prevent potential complications and ensure favorable outcomes.

## Introduction

Pleomorphic adenoma (PA) is the most prevalent benign salivary gland tumor type, typically developing slowly and without symptoms [1]. It can be seen in people of all age groups but is more common in women and individuals aged 30-60. The etiological factors contributing to PA are mainly unknown; however, some studies suggest that the oncogenic simian virus (SV40) and Epstein-Barr virus (EBV), as well as genetic factors including PLAG1 and HMGA2, may play a role in the onset or progression [1]. On a microscopic level, PA appears as a combination of polygonal epithelial and spindle-shaped myoepithelial cells within a varied stroma containing different types of elements like mucoid, myxoid, cartilaginous, or hyaline materials [1]. The epithelial cells have the ability to create tube-like formations, layers, groupings, or twisting patterns, frequently encircled by myoepithelial layers merging with the neighboring stroma. The tumor does not have a genuine capsule but instead is surrounded by a fibrous pseudo capsule that varies in thickness [1]. PA diagnosis typically relies on the findings from fine-needle aspiration (FNA), ultrasonography (USG), and computed tomography (CT) scan results [2]. PAs makeup 66% of all tumors in the salivary glands and commonly impact the superficial lobe of the parotid gland in 85% of instances [2]. However, in rare cases, they can also occur outside of the parotid gland in areas such as the nasal cavity. PAs have an incidence rate of only 0.4% in the nasal cavity and present diagnostic and surgical challenges due to their rarity and the complex anatomy of the nose [3]. Recognizing PA in atypical locations is crucial for timely intervention and management to prevent complications such as recurrence or malignant transformation. Differential diagnoses in such cases include other benign and malignant tumors like adenocarcinoma, mucoepidermoid carcinoma, and olfactory neuroblastoma, which necessitates careful histopathological evaluation to ensure accurate diagnosis and appropriate treatment. PA of the nasal cavity is a rare clinical observation, which we report here.

## Case presentation

**Figure 1:**
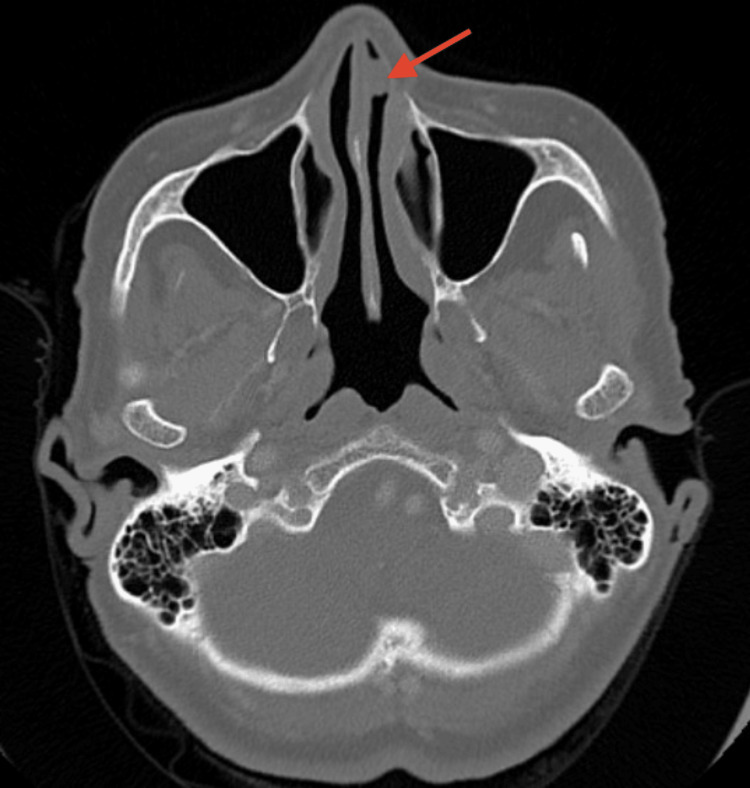
Axial CT scan revealing the presence of an 8x8 mm mass in the left nasal cavity CT, computed tomography

**Figure 2:**
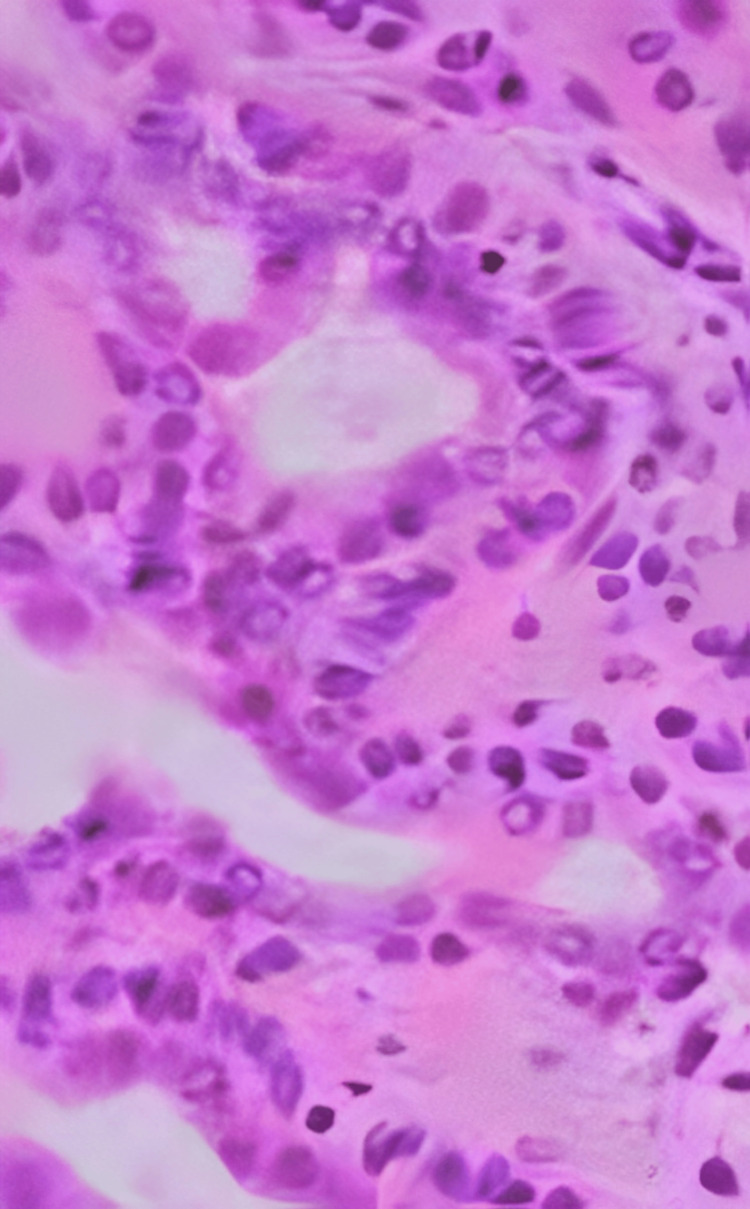
Histological presentation of PA PA, pleomorphic adenoma

An 87-year-old female patient presented to the clinic with chronic left-sided nasal symptoms, including rhinorrhea, postnasal drip, and acid reflux. The patient's vitals were stable, with a heart rate of 76 bpm and blood pressure of 126/70. Her medical history included high cholesterol, hypertension, glaucoma, neuropathy, anxiety, and tachycardia. There was no significant family history of nasal or salivary gland tumors but she did endorse an allergic reaction to Flonase nasal spray. She reported nasal discomfort in her left nostril, reduced nasal patency, and leakage of medications (Atrovent 0.03% and saline spray) from her left nostril upon administration. A rhinoscopy and CT scan revealed an 8x8 mm fleshy mass in the roof of the nasal vestibule within the left nostril (Figure 1). Given the patient's age, overall health, and the location of the mass, general anesthesia was chosen to ensure patient comfort and optimal surgical conditions. The decision for surgical excision was based on the mass's potential for obstruction and the need for histopathological diagnosis. Under general anesthesia, an excision of the 8x8 mm left anterior nasal mass was performed, with the base of the pedicle cauterized using electrocautery to minimize bleeding and reduce the risk of recurrence. The specimen was sent to pathology, revealing the mass to be a biphasic neoplasm consistent with a PA (Figure 2). Post-operatively, the patient reported significant improvement in her nasal symptoms, including reduced discomfort and improved nasal patency. A follow-up schedule was established every three months to monitor for recurrence, given the potential for regrowth or complications associated with PAs.

## Discussion

PAs within the nasal cavity show a higher presence of epithelial components than stromal elements in comparison to tumors found in the main salivary glands [4]. Analysis using immunohistochemistry shows the presence of cytokeratins, S100 protein, GFAP, Vimentin, and SMA staining, indicating the tumor's mixed epithelial and stromal composition [4]. The differential diagnosis for intra-nasal PA includes a wide range of benign and malignant tumors like squamous cell carcinoma, adenocarcinoma, adenoid cystic carcinoma, mucoepidermoid carcinoma, melanoma, olfactory esthesioneuroblastoma, polyps, papillomas (including inverted papilloma), angiofibromas, and osteomas. The rarity and unique histological characteristics of neuroesthesioepithelioma can make diagnosis challenging, including small cell growth in rosette formations and positive S100 protein staining [4]. Surgical excision is the main method used to treat PAs. It is crucial to completely remove the tumor to prevent malignant transformation into carcinoma ex PA, a possibility in 4% of cases [5]. More specifically, around 2.4% of nasal cavity PAs have the potential to become cancerous. The post-surgery recurrence rate is about 7.5%, underscoring the importance of complete removal to reduce positive margins, capsule rupture, and tumor spillage, which are the main factors leading to recurrence [6]. The surgical methods used depend on the size, location, and extent of the tumor, which can include lateral rhinotomy, trans-nasal or mid-facial degloving, or intra-nasal excision. Due to the small size of the PA, we proceeded with intra-nasal excision. Potential surgical complications, such as bleeding, infection, and damage to surrounding structures, were carefully managed through meticulous surgical technique and postoperative monitoring. The patient in this case had an uneventful recovery, with no immediate complications observed. When there are positive surgical margins, postoperative radiation therapy (RT) is thought to improve local control [5]. Nevertheless, the decision to use RT is still controversial because PA is usually not aggressive, so it is only considered for cases with recurring problems. Factors like how quickly the cancer comes back, the age of the patient, and how well the first surgery removed all of the tumor influence the choice to give RT [7]. Our follow-up protocol is carefully crafted to observe for any signs of recurrence including malignant transformation and address any developing symptoms. A complete understanding of malignant transformation mechanisms is lacking; however, markers such as MUC1, HMGIC amplification, and MDM2 overexpression could hint at the risk of recurrence [8]. The prognosis for carcinoma ex PA differs based on invasiveness; smaller tumors have a better outlook, while larger tumors have a poorer prognosis, with a 30% five-year survival rate [4]. Histological assessment is still the preferred method for differentiating between PA and carcinoma ex PA, which helps in determining the proper treatment and prognosis. The histological features of the nasal cavity PA in this case, characterized by a biphasic pattern of epithelial and myoepithelial cells within a fibromyxoid stroma, were consistent with typical PAs of the salivary glands. However, the higher epithelial component observed aligns with findings in nasal cavity PAs, differentiating them from their salivary gland counterparts, which often have a more pronounced stromal component. Understanding these differences is critical for accurate diagnosis and treatment planning.

## Conclusions

PA can present unique challenges when occurring in non-classical locations such as the nasal cavity. In our patient, the successful excision of the adenoma not only alleviated her symptoms but also mitigated the risk of complications associated with the growth of such tumors in a confined anatomical space. Furthermore, surgical intervention of such masses is needed to avoid complications associated with malignant transformations or local effects. Future research should focus on improving diagnostic techniques for early detection of PA in atypical locations and exploring additional etiological factors, which may provide insights into the pathogenesis and prevention of these tumors. Additionally, long-term follow-up and continuous monitoring are essential to prevent recurrence and manage any arising complications effectively. This case highlights the need for heightened awareness among physicians regarding the presentation of PA in unusual anatomical sites and the importance of comprehensive management strategies.
